# Physiological presentation and risk factors of long COVID in the UK using smartphones and wearable devices: a longitudinal, citizen science, case–control study

**DOI:** 10.1016/S2589-7500(24)00140-7

**Published:** 2024-08-12

**Authors:** Callum Stewart, Yatharth Ranjan, Pauline Conde, Shaoxiong Sun, Yuezhou Zhang, Zulqarnain Rashid, Heet Sankesara, Nicholas Cummins, Petroula Laiou, Xi Bai, Richard J B Dobson, Amos A Folarin

**Affiliations:** a Department of Health Informatics and Biostatistics, Institute of Psychiatry, Psychology and Neuroscience, King's College London, London, UK; b Department of Computer Science, University of Sheffield, Sheffield, UK; c Institute of Health Informatics, University College London, London, UK; d NIHR Maudsley Biomedical Research Centre, South London and Maudsley NHS Foundation Trust, London, UK

## Abstract

**Background:**

The emergence of long COVID as a COVID-19 sequela was largely syndromic in characterisation. Digital health technologies such as wearable devices open the possibility to study this condition with passive, objective data in addition to self-reported symptoms. We aimed to quantify the prevalence and severity of symptoms across collected mobile health metrics over 12 weeks following COVID-19 diagnosis and to identify risk factors for the development of post-COVID-19 condition (also known as long COVID).

**Methods:**

The Covid Collab study was a longitudinal, self-enrolled, community, case–control study. We recruited participants from the UK through a smartphone app, media publications, and promotion within the Fitbit app between Aug 28, 2020, and May 31, 2021. Adults (aged ≥18 years) who reported a COVID-19 diagnosis with a positive antigen or PCR test before Feb 1, 2022, were eligible for inclusion. We compared a cohort of 1200 patients who tested positive for COVID-19 with a cohort of 3600 sex-matched and age-matched controls without a COVID-19 diagnosis. Participants could provide information on COVID-19 symptoms and mental health through self-reported questionnaires (active data) and commercial wearable fitness devices (passive data). Data were compared between cohorts at three periods following diagnosis: acute COVID-19 (0–4 weeks), ongoing COVID-19 (4–12 weeks), and post-COVID-19 (12–16 weeks). We assessed sociodemographic and mobile health risk factors for the development of long COVID (defined as either a persistent change in a physiological signal or self-reported symptoms for ≥12 weeks after COVID-19 diagnosis).

**Findings:**

By Aug 1, 2022, 17 667 participants had enrolled into the study, of whom 1200 (6·8%) cases and 3600 (20·4%) controls were included in the analyses. Compared with baseline (65 beats per min), resting heart rate increased significantly during the acute (0·47 beats per min; odds ratio [OR] 1·06 [95% CI 1·03–1·09]; p<0·0001), ongoing (0·99 beats per min; 1·11 [1·08–1·14]; p<0·0001), and post-COVID-19 (0·52 beats per min; 1·04 [1·02–1·07]; p=0·0017) phases. An increased level of historical activity in the period from 24 months to 6 months preceding COVID-19 diagnosis was protective against long COVID (coefficient –0·017 [95% CI –0·030 to –0·003]; p=0·015). Depressive symptoms were persistently elevated following COVID-19 (OR 1·03 [95% CI 1·01–1·06]; p=0·0033) and were a potential risk factor for developing long COVID (1·14 [1·07–1·22]; p<0·0001).

**Interpretation:**

Mobile health technologies and commercial wearable devices might prove to be a useful resource for tracking recovery from COVID-19 and the prevalence of its long-term sequelae, as well as representing an abundant source of historical data. Mental wellbeing can be impacted negatively for an extended period following COVID-19.

**Funding:**

National Institute for Health and Care Research (NIHR), NIHR Maudsley Biomedical Research Centre, UK Research and Innovation, and Medical Research Council.

## Introduction

Despite the development and successful roll-out of vaccinations and treatments across the UK, COVID-19 remains a threat to individual and public health in terms of the acute illness, risk of death, long-term illness following infection, and the development of new variants.^1^ Persistent symptoms following SARS-CoV-2 infection, often termed post-COVID-19 condition (also known as long COVID), are thought to affect a considerable number of patients. The presence of these long-term symptoms has been largely illuminated through subjective accounts from people with the condition.^2^ Although there has been an influx of research starting to address the prevalence, clinical features, and risk factors^3^ of long COVID, our understanding of the condition remains sparse.

Attempts have been made to categorise long COVID on the basis of symptoms, time periods, and causes; however, it remains a loosely defined syndrome with multiple associated terms.^4^ In the UK, the National Institute for Health and Care Excellence (NICE) suggests acute COVID-19 for symptoms lasting up to 4 weeks, ongoing symptomatic COVID-19 for symptoms lasting from 4 to 12 weeks, and post-COVID-19 syndrome for symptoms continuing for longer than 12 weeks.^5^ Symptoms have been reported across various organs and body systems, including cardiorespiratory, neurological, psychological, muscular, gastrointestinal, and systemic.^6^, ^7^ Common symptoms include fatigue, dyspnoea, anxiety, sleep disorders, pain, dizziness, and anosmia.^8^ Due in part to differences between study cohorts, terminology, and study design, prevalence estimates for long COVID vary from 0% to as high as 93%.^9^ Many studies recruit participants from hospitalised populations; therefore, patients with severe COVID-19 are generally selected, which might increase the perceived incidence of long COVID.

**Research in context**

**Evidence before this study**

Digital health technologies have provided another modality to understand and monitor disease and are particularly suited to chronic conditions that require long-term monitoring. We searched PubMed, without language restrictions, from the date of database inception to July 1, 2022, for studies on post-COVID-19 condition (also known as long COVID) that used data from wearable devices or mobile health technologies using the following string of search terms: “((COVID* OR SARS-COV-2) AND (long OR persistent OR hauler OR post OR sequelae)) AND (mHealth OR wearable OR telemedicine OR app)”. Of the 2144 results, most returned studies concerned the role of telemedicine in the delivery of care for other conditions from the start of the COVID-19 pandemic. Several studies related to the monitoring, detection, or diagnosis of acute COVID-19 (0–4 weeks after diagnosis). Other studies used digital technology or telemedicine to treat COVID-19 or to assess the effect of rehabilitation courses. Eight studies investigated long COVID through remote digital technologies, of which six used data from self-reported questionnaires collected through televisits or apps to characterise symptoms and trajectories of long COVID. One study was a large-scale community study from the ZOE COVID Symptom app. Three studies used passively collected data from commercial or experimental wearable sensors to describe changes in heart rate, sleep duration, and physical activity in a population of patients with COVID-19 following acute infection. Two studies showed a pattern of bradycardia and tachycardia in resting heart rate, with a persistent change in some cases lasting over 4 months.

**Added value of this study**

This longitudinal, self-enrolled, community, case–control study provides a unique viewpoint for quantifying the features and risk factors of long COVID. This study incorporates survey data alongside pervasive data from wearable devices, including long-term historical data from the pre-pandemic period. Self-reported questionnaires included regular mental health measures and physical symptoms related to COVID-19. The study population was recruited remotely and openly throughout the pandemic and, therefore, included non-hospitalised participants and those with a mild response to acute COVID-19. Data were often collected before the date of COVID-19 diagnosis, which did not rely on participant recall and the inclusion of historical data from wearable devices from before study enrolment. Software and data collection infrastructure have been openly sourced to facilitate the reuse of this system for future digital epidemiology research or monitoring programmes.

**Implications of all the available evidence**

Acute and persistent effects following COVID-19 are visible in data streams from wearable devices and mobile health technologies, and these patterns have been substantiated in this study and similar studies conducted worldwide. A low historical level of physical activity seems to be an important risk factor for developing long COVID. A subset of participants showed reduced mental wellbeing following COVID-19, and previous mental health issues might also be predictive of developing long. Personal wearables, which are becoming increasingly common, offer an objective and often pre-existing measurement of activity and health that could be further used in monitoring infection and recovery from COVID-19. Incorporation of wearables in clinical care could widen the information available to clinicians and increase the velocity of data sampling in long lasting and novel diseases, such as long COVID.

The COVID-19 pandemic has been a focal point for the increased emergence of digital health technologies in research and health care. Multiple studies have used digital health approaches to better understand trajectories of, diagnose, and estimate the prevalence of COVID-19 and its long-term sequelae. Mobile health modalities can offer insight that is complementary to traditional techniques for the treatment of long COVID.

Passive mobile sensing refers to data collection from participants' mobile devices, which does not involve direct user action. It can provide an objective, continuous, and scalable measure of health. Additionally, long periods of historical data are often available from fitness devices. The availability of wearable data outside of medical care pathways offers an avenue to observe people who might otherwise be missed. For example, through the use of commercial data from wearable devices, clinically significant morbidity has been shown in people with influenza who do not seek medical care.^10^

Covid Collab is an observational mobile health study that began in June, 2020.^11^ Participants were enrolled through the study app, Mass Science, and were prompted to complete regular surveys on COVID-19 symptoms, vaccination status, and diagnosis status, as well as mental wellbeing before, throughout, and after infection. The app was available worldwide, but the vast majority of recruitment was conducted in the UK. Participants could share existing and prospective data from their Fitbit accounts through the RADAR-Base platform.^12^ In the current study, we retrieved data from these wearable devices covering a period before the pandemic, which provided historical information on participants.

In this study, we aimed to quantify the prevalence and severity of long-term symptoms across collected mobile health metrics, consisting of heart rate, heart rate variability, sleep, physical activity, self-reported symptoms, and mood. We also aimed to identify risk factors for the severity and duration of persistent symptoms. Accordingly, in this Article, long COVID was considered as persistent changes or symptoms at and beyond 12 weeks following COVID-19 diagnosis.

## Methods

### Study design and participants

In this longitudinal, self-enrolled, community, case–control study, we recruited participants from across the UK through a smartphone app,^11^ media publications, and promotion within the Fitbit app between Aug 1, 2020, and May 31, 2021. Adults (aged ≥18 years) who reported a COVID-19 diagnosis with a positive antigen or PCR test before Feb 1, 2022, were eligible for inclusion. Participants provided electronic consent through the app to take part in this study.

Our changing understanding of COVID-19, the pandemic, and requirements of this study led to some amendments to the protocol, including an extended sociodemographic questionnaire introduced on Nov 17, 2020, building on the initial registration questionnaire. A detailed description of the study is available in a protocol paper.^11^ Additionally, participants were able to donate data from various sources, such as geolocation or wearable devices, and were free to provide as much data as they chose. Therefore, there are differences in data availability between participants. This study was approved by King's College London's Psychiatry, Nursing, and Midwifery Research Ethics Panel (LRS-18/19-8662) and is reported in accordance with STROBE guidelines.

### Data collection

There were two major categories of data in this study: passive and active. Active data were responses to questionnaires delivered in the app and consisted of the eight-item Patient Health Questionnaire (PHQ-8) depression scale,^13^ the seven-item assessment of Generalised Anxiety Disorder (GAD-7) scale,^14^ a scale for arousal and valence,^15^ and a questionnaire on COVID-19 symptoms. Additionally, participants could optionally submit COVID-19 diagnoses and vaccination events.

Passive data were collected from instruments that did not require conscious participant involvement. These data comprised daily summaries of heart rate, heart rate variability, sleep duration, step count, and activity logs provided through the participant's sharing of data from commercial wearable devices (all models of Fitbit; Google Fitbit, San Francisco, CA, USA). Participants could freely enter symptoms, but particular symptoms were prompted for directly (ie, anosmia, fatigue, muscle pain, headache, nausea, confusion, cough, and breathing problems). Each symptom was rated in severity as none, mild, moderate, or severe.

### Statistical analysis

We compared a cohort of 1200 patients who tested positive for COVID-19 (COVID-19 cohort), reported a diagnosis, and met the Fitbit data completeness criteria, with a cohort of 3600 matched controls (control cohort) using statsmodels (Python).^16^ For each case participant, three participants with the same sex, within 2 years of age, and without a COVID-19 diagnosis were randomly selected without replacement. A description of the matching algorithm is available in the appendix (p 5). The cohorts were retrospectively tested for balance with a Chi square test of independence across other sociodemographic factors. Data from each control were taken from the period surrounding the matched cases' infection date of COVID-19 (1–16 weeks after).

As derived from NICE guidelines,^5^ each metric was aggregated over a period of 4 weeks from the start of each phase: acute COVID-19 (0–4 weeks), ongoing COVID-19 (4–8 weeks), and post-COVID-19 (12–16 weeks). Group-wide resting heart rate, heart rate variability, sleep duration, sleep efficiency, step count, PHQ-8 score, GAD-7 score, and self-rated valence and arousal were the metrics compared between periods of acute COVID-19, ongoing COVID-19, and post-COVID-19, following a self-reported PCR or antigen diagnosis of COVID-19. For each time period and metric, we performed a logistic regression between cases and controls, accounting for age, sex, BMI, ethnicity, children, and current smoking status.

To test risk factors for long COVID, it was necessary to define a candidate group of participants who were likely to have long COVID based on the data that we had available. We considered the following two approaches. First, we used the change in resting heart rate at 12 weeks after COVID-19 diagnosis as a proxy for long COVID, whereby a greater change compared with baseline resting heart rate indicated a more likely case of developing mild or severe long COVID. As such, we estimated the resting heart rate that a participant would be expected to have without COVID-19 by fitting a Bayesian structural time series model to each participant's resting heart rate until diagnosis using the CausalImpact library on GitHub.^17^ This model comprised a local-level model, a seasonal model with a period of 28 days, and a regularised regression on a set of 500 participants who were not otherwise involved in the analysis. The difference between the estimated and actual resting heart rate at 12 weeks was used as the outcome in a multiple linear regression, with age, sex, historical activity, historical sleep duration, and resting heart rate change in the acute period as predictors. Historical activity was the mean duration of time in minutes spent in the Fitbit's high activity level per day. Sleep duration was the mean time spent in minutes asleep per day. The historical period was considered between 2 years and 6 months before the diagnosis of COVID-19. The baseline to acute change in resting heart rate was defined as the difference between the mean resting heart rate 1–4 weeks before a COVID-19 diagnosis and up to 4 weeks after a COVID-19 diagnosis. Participants were included if a baseline resting heart rate could be established in the month before diagnosis, if there was at least 4 days of data on resting heart rate at week 12, and if they had at least 7 days' worth of data on sleep duration and activity in the historical period.

Second, we considered participants who self-reported symptoms for an extended period following a self-reported case of COVID-19 (ie, reported through an in-app questionnaire). The self-reported symptoms submitted by all participants who reported a positive diagnosis were used to establish length of illness and to divide the cohort of participants with COVID-19 into short and long COVID groups. If at least one symptom was reported at least once per week for at least 12 weeks, the participant was assigned to the symptom-based long COVID group. Participants were otherwise assigned to the symptom-based short COVID-19 group. We performed logistic regressions to classify both of these groups based on demographics, passive data, and mental health data at baseline and during the acute phase.

The inclusion criteria for continuous variables were based on a completion rate of at least 60% at baseline and in the acute phase. p values were adjusted with the Benjamini–Hochberg procedure.^18^

### Role of the funding source

The funders of the study had no role in study design, data collection, data analysis, data interpretation, or writing of the report.

## Results

By Aug 1, 2022, 17 667 participants had enrolled into the study, of whom 1200 (6·8%) cases and 3600 (20·4%) controls were included in the analyses. Most participants were recruited from within the UK (16 748 [95%]) and were women (table 1). In the COVID-19 cohort, 573 (47·8%) participants completed the extended sociodemographic questionnaire. Different numbers of participants were included in different aspects of the analysis due to differing rates of data completion across modalities.

**Table 1:** Sociodemographic characteristics of COVID-19 and control cohorts

		**COVID-19 cohort**	**Control cohort**	**p value**
**Initial questionnaire**				
Number completed		1200 (100%)	3600 (100%)	..
Sex				
	Female	894 (74·5%)	2682 (74·5%)	1·00
	Male	306 (25·5%)	918 (25·5%)	..
Age, years		44·38 (12·93); 18·00–87·00	44·80 (12·96); 18·00–88·00	0·84
Height, cm		168·51 (9·52); 124·40–198·10	168·84 (9·66); 121·90–203·00	0·74
Weight, kg		79·01 (19·13); 26·50–178·20	77·68 (18·45); 31·70–187·00	0·16
Smoking status				
	Never	680 (56·7%)	2125 (59·0%)	..
	Former	331 (27·6%)	993 (27·6%)	0·53
	Current	169 (14·1%)	473 (13·1%)	..
	Data missing	20 (1·7%)	9 (0·3%)	..
**Extended questionnaire**				
Number completed		573 (47·8%)	1854 (51·5%)	..
Comorbidities				
	Asthma	126 (22·0%)	360 (19·4%)	0·20
	Hypertension	57 (9·9%)	173 (9·3%)	0·72
	Diabetes	22 (3·8%)	69 (3·7%)	1·00
	Depression	135 (23·6%)	477 (25·7%)	0·32
	Anxiety	101 (17·6%)	342 (18·4%)	0·70
Employment status				
	Full-time	322 (56·2%)	1001 (54·0%)	..
	Part-time	81 (14·1%)	280 (15·1%)	..
	Retired	68 (11·9%)	227 (12·2%)	0·44
	Student	15 (2·6%)	79 (4·3%)	..
	Unemployed	17 (3·0%)	55 (3·0%)	..
Marital status				
	In a relationship	452 (78·9%)	1412 (76·2%)	..
	Single or separated	118 (20·6%)	421 (22·7%)	0·23
	Unknown	3 (0·5%)	21 (1·1%)	..
Living situation				
	Alone	48 (8·4%)	242 (13·1%)	..
	With a partner	339 (59·2%)	1171 (63·2%)	<0·0001
	With family	205 (35·8%)	484 (26·1%)	..
	House share	12 (2·1%)	58 (3·1%)	..
	With children (aged <18 years)	158 (27·6%)	403 (21·7%)	0·0045
	With adult children	86 (15·0%)	183 (9·9%)	0·0008
Ethnicity				
	White	512 (89·4%)	1686 (90·9%)	..
	Asian	15 (2·6%)	35 (1·9%)	..
	Black	4 (0·7%)	15 (0·8%)	..
	Mixed race	1 (0·2%)	4 (0·2%)	0·83
	Other	2 (0·3%)	5 (0·3%)	..
	Unknown	39 (6·8%)	108 (5·8%)	..

Data are n (%) or mean (SD); range.

**Figure 1:**
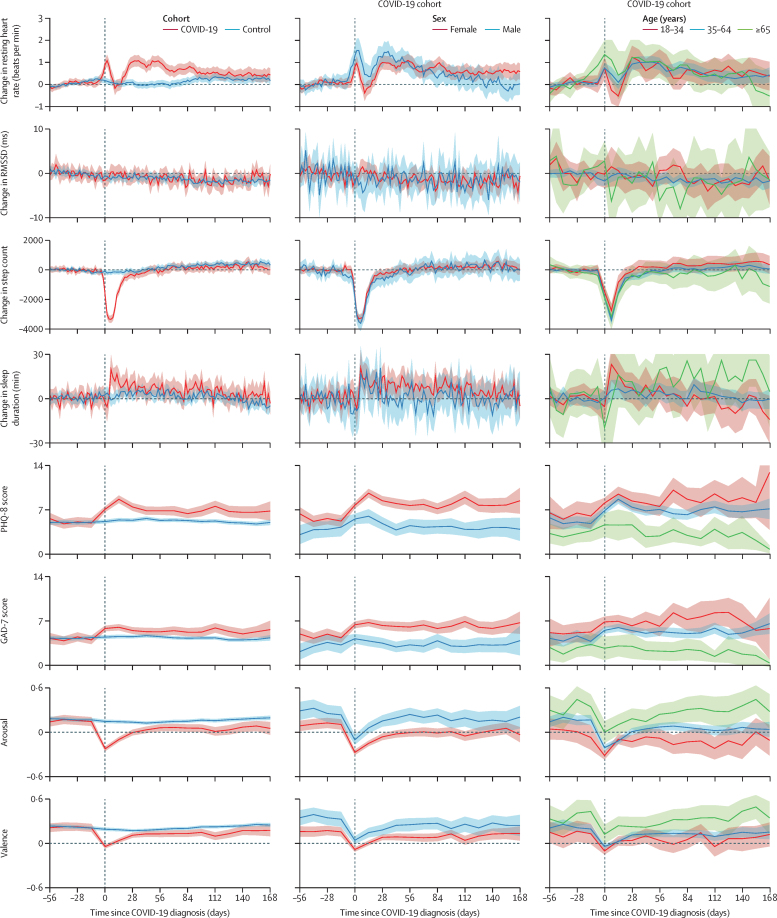
Physiological signals and self-reported measures of mental health over time Metrics plotted by cohort, by sex in the COVID-19 cohort, and by age group in the COVID-19 cohort. Dates range from 8 weeks before to 24 weeks after COVID-19 diagnosis. Physiological signals (resting heart rate, RMSSD, step count, and sleep duration) measured through wearable devices (passive data) and self-reported measures (PHQ-8 score, GAD-7 score, arousal, and valence) through survey responses (active data). References for changes in physiological signals are baseline values. Arousal and valence scores are reported on a visual analogue scale ranging from –1 to +1. The shaded area corresponds to 95% CIs over a 14-day window for self-reported measures, a 1-day window for physiological signals by cohort and sex, and a 7-day window for physiological signals by age group. GAD-7=seven-item assessment of Generalised Anxiety Disorder. PHQ-8=eight-item Patient Health Questionnaire. RMSSD=root mean square of successive differences between heartbeats.

Resting heart rate among cases increased significantly in every period (table 2). However, due to bidirectional (ie, increasing and decreasing over time) acute changes in this metric (figure 1A), the difference during the acute period (0·47 beats per min; odds ratio [OR] 1·06 [95% CI 1·03–1·09]; p<0·0001) was less than the ongoing period (0·99 beats per min; 1·11 [1·08–1·14]; p<0·0001) and similar to the post-COVID-19 period (0·52 beats per min; 1·04 [1·02–1·07]; p=0·0017). Step count was negatively affected during the acute period of COVID-19 and more modestly in both subsequent periods. Sleep duration did not differ significantly between cohorts (table 2).

**Table 2:** Adjusted group-wide logistic regression during acute, ongoing, and post-COVID-19 periods

	**Acute COVID-19**		**Ongoing COVID-19**		**Post-COVID-19**	
	OR (95% CI)	p value	OR (95% CI)	p value	OR (95% CI)	p value
Resting heart rate, beats per min	1·06 (1·03–1·09)	<0·0001*	1·11 (1·08–1·14)	<0·0001*	1·04 (1·02–1·07)	0·0017*
Root mean square of successive differences between heartbeats	1·00 (0·74–1·35)	0·99	1·10 (0·82–1·47)	0·54	1·01 (0·67–1·50)	0·97
Step count, per 1000	0·86 (0·84–0·88)	<0·0001*	0·96 (0·94–0·98)	<0·0001*	0·98 (0·96–0·99)	0·011*
Sleep efficiency	0·99 (0·98–1·00)	0·27	1·00 (0·99–1·01)	0·79	0·99 (0·98–1·01)	0·36
Sleep duration, mins	1·00 (1·00–1·00)	0·50	1·00 (1·00–1·00)	0·69	1·00 (1·00–1·00)	0·52
PHQ-8 score	1·07 (1·05–1·09)	<0·0001*	1·05 (1·03–1·07)	<0·0001*	1·03 (1·01–1·06)	0·0033*
GAD-7 score	1·02 (1·00–1·04)	0·0078	1·02 (1·00–1·04)	0·070	1·03 (1·01–1·06)	0·020*
Arousal	0·16 (0·13–0·20)	<0·0001*	0·52 (0·40–0·66)	<0·0001*	0·64 (0·48–0·84)	0·0014*
Valence	0·29 (0·23–0·37)	<0·0001*	0·65 (0·51–0·84)	0·0009*	0·75 (0·56–1·01)	0·062

Group-wide logistic regression between COVID-19 and control cohorts adjusted for age, sex, BMI, ethnicity, children, and smoking status during acute COVID-19 (<4 weeks after diagnosis), ongoing COVID-19 (4–8 weeks), and post-COVID-19 (>12 weeks). Resting heart rate, sleep efficiency, sleep duration, and step count were calculated relative to baseline values taken as a mean value 12 weeks before COVID-19 diagnosis. OR=odds ratio. PHQ-8=eight-item patient health questionnaire. GAD-7=seven-item assessment of generalised anxiety disorder.

* Significant (α<0·05) after adjustment with the Benjamini–Hochberg procedure.

Self-reported measures of depression and arousal were significantly negatively affected during every period, although the difference between groups decreased over time from a difference of +2·16 in the acute phase (OR 1·07 [95% CI 1·05–1·09]; p<0·0001) to +0·83 in the post-COVID-19 phase (1·03 [1·01–1·06]; p=0·0033) in mean PHQ-8 score. Additionally, anxiety was significantly increased during the acute phase (OR 1·02 [95% CI 1·00–1·04]; p<0·0001) and post-COVID-19 phase (1·03 [1·01–1·06]; p=0·020). No significant differences were found in any metric between the COVID-19 and control cohorts in the period from 8 weeks to 4 weeks before COVID-19 diagnosis.

In total, 597 (49·8%) of participants in the COVID-19 cohort were included in the multiple linear regression analysis that was conducted to establish whether historical data from a fitness wearable before a COVID-19 diagnosis was a risk factor for persistent elevated resting heart rate at 12 weeks after diagnosis (table 3). Increased historical activity (ie, the time spent taking part in heavy activity as measured by a wearable device) was negatively correlated with risk of developing long COVID (–0·017 [95% CI –0·030 to –0·003]; p=0·015), suggesting a slightly protective effect against long COVID for more active people. A non-significant positive trend was shown between female sex and risk of developing long COVID.

**Table 3:** Regression of risk factors for long COVID

	**Coefficient**	**SE**	**95% CI**	**p value**
Intercept	−1·989	1·078	−4·097 to 0·139	0·067
Age	0·027	0·011	0·005 to 0·050	0·017*
Female sex	0·558	0·337	−0·104 to 1·219	0·098
Historical activity	−0·017	0·007	−0·030 to −0·003	0·015*
Historical sleep	0·003	0·002	−0·001 to 0·007	0·160
Resting heart rate†	0·281	0·039	0·204 to 0·357	<0·0001*

Data collected passively through wearable devices (except for age and sex).

* Significant (α<0·05*)* after adjustment with the Benjamini–Hochberg procedure.

† During the acute COVID-19 phase.

**Figure 2:**
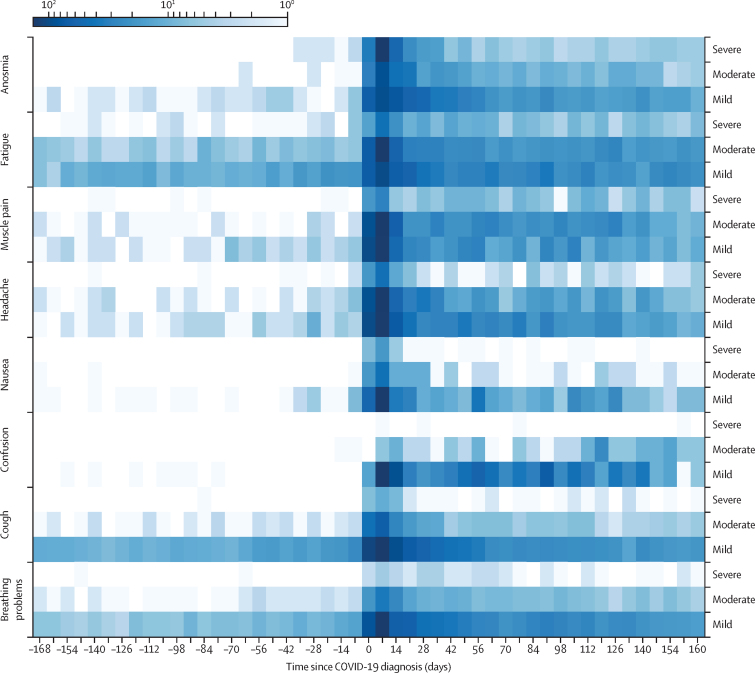
A heatmap of prompted self-reported symptoms The heatmap represents counts of self-reported symptom severity among patients in the COVID-19 cohort 24 weeks before and after the date of diagnosis. Although it was possible to input additional symptoms, these symptoms were specifically prompted for in the survey. The colour intensity indicates the number of participants who reported a symptom (log scale). Severity could be reported on a three-point scale (mild, medium, or severe).

All symptoms showed an increase in prevalence and severity at the point of COVID-19 diagnosis, often with an elevated level at 24 weeks (figure 2). Some symptoms—namely, fatigue, cough, and breathing problems—had an increased prevalence at baseline. Fatigue persisted with moderate to high severity to 24 weeks. Coughing and breathing problems also persisted for 24 weeks, but with mild severity (figure 2).

A series of multiple logistic regressions were performed to establish the likelihood of having long COVID based on the effects of covariates from sociodemographic data, wearable devices, and the mental health survey (table 4). Of the participants who tested positive for COVID-19, 505 (42·1%) were included after exclusion of participants with missing data on covariates: 396 (78·4%) in the symptom-based short COVID-19 group and 109 (21·6%) in symptom-based long COVID-19 group.

**Table 4:** Adjusted logistic regression analysis for variables across symptom-based short and long COVID cohorts

		**Total number of patients (number of patients with long COVID)**	**OR (95% CI)**	**p value**
Age		505 (109)	1·03 (1·01–1·05)	0·0005*
BMI		505 (109)	1·01 (0·97–1·04)	0·75
Female sex†		505 (109)	1·13 (0·69–1·87)	0·63
Black, Asian, or other ethnicity‡		505 (109)	1·07 (0·38–2·96)	0·902
Age range, years§				
	30–40	505 (109)	5·13 (1·13–23·27)	0·034
	41–50	505 (109)	7·03 (1·61–30·78)	0·0096*
	51–60	505 (109)	9·20 (2·14–39·62)	0·0029*
	>60	505 (109)	9·47 (2·14–41·87)	0·0030*
Comorbidity				
	Asthma	505 (109)	1·43 (0·87–2·36)	0·16
	Hypertension	505 (109)	1·47 (0·71–3·02)	0·30
	Diabetes	505 (109)	1·43 (0·87–2·36)	0·16
Smoking status¶				
	Current	505 (109)	0·95 (0·39–2·30)	0·904
	Former	505 (109)	0·82 (0·51–1·33)	0·42
Step count, per 1000				
	Baseline	397 (89)	1·0 (1·00–1·00)	0·96
	Acute phase	354 (83)	1·0 (1·00–1·00)	0·76
Resting heart rate, beats per min				
	Baseline	274 (65)	1·03 (0·99–1·07)	0·11
	Acute phase	263 (63)	1·04 (1·00–1·08)	0·060
Activity, mins				
	Baseline	320 (67)	1·00 (0·99–1·02)	0·42
	Acute phase	291 (67)	1·00 (0·99–1·01)	0·92
Sleep duration, mins				
	Baseline	221 (72)	1·00 (1·00–1·01)	0·046
	Acute phase	230 (74)	1·00 (1·00–1·00)	0·37
Sleep efficiency				
	Baseline	221 (72)	0·98 (0·96–1·01)	0·18
	Acute phase	230 (74)	0·99 (0·97–1·01)	0·31
PHQ-8 score				
	Baseline	177 (44)	1·14 (1·07–1·22)	<0·0001*
	Acute phase	357 (85)	1·08 (1·04–1·13)	0·0006*
GAD-7 score				
	Baseline	183 (43)	1·12 (1·05–1·21)	0·0014*
	Acute phase	353 (85)	1·06 (1·01–1·11)	0·015
Symptom severity during acute phase		343 (81)	0·95 (0·88–1·03)	0·22
Number of symptoms during acute phase		343 (81)	0·89 (0·77–1·03)	0·13

ORs with 95% CIs adjusted for age, gender, and ethnicity. OR=odds ratio.

* Significant (α<0·05*)* after adjustment with the Benjamini–Hochberg procedure.

† Reference sex: male.

‡ Reference ethnicity: White.

§ Reference age range: 10–30 years.

¶ Reference smoking status: never.

Discretised age ranges were used to assess the factor of age on developing long COVID and age was the most prominent risk factor. Participants aged 40–50 years (OR 7·03 [95% CI 1·61–30·78]; p=0·0096), 50–60 years (9·20 [2·14–39·62]; p=0·0029), and older than 60 years (9·47 [2·14–41·87]; p=0·0030) were at significantly higher risk of developing long COVID than were those aged 18–30 years (table 4). The comorbidities tested, asthma, hypertension, and diabetes, did not show a significant effect on the risk of developing long COVID; however, the ORs were all greater than one (table 4). PHQ-8 and GAD-7 scores at baseline were significantly associated with the risk of developing long COVID (1·14 [1·07–1·22]; p<0·0001 and 1·12 [1·05–1·21]; p=0·0014), as were PHQ-8 scores during the acute phase (1·08 [1·04–1·13]; p=0·0006).

## Discussion

In this longitudinal, self-enrolled, community, case–control study, we investigated persistent symptoms of and recovery from COVID-19 using mobile health data. We found a signal for long COVID in metrics from both passive and active data collection, as well as associations between psychological and physiological metrics and behaviour before COVID-19 diagnosis.

We found that several metrics measured with wearable devices and mental health surveys changed significantly in the COVID-19 cohort during the acute phase of infection, some of which remained significantly different from the control cohort for longer than 12 weeks. Resting heart rate was the metric from a wearable device with the longest lasting noticeable change. Our estimate that 7·0% of participants (84 of 1200) had a long-term change in heart rate coinciding with COVID-19 is somewhat less than the estimate of 13·7% of participants (32 of 234) in another study that reported an increase in resting heart rate of at least 5 beats per min at 12 weeks.^19^ The Bayesian structural time series model accounting for seasonal effects and the effect of lockdown might help to explain the difference. Self-rated depression and arousal remained negatively affected in the post-COVID-19 phase. The increase in self-reported anxiety and high variance suggest a subset of people who test positive for COVID-19 have persistent symptoms of depression and low energy for at least 12 weeks. As previously reported,^19^ we observed a pattern of initially increased resting heart rate, followed by reduced resting heart rate from week 2 to week 3, and finally a chronic or long-lasting increase in resting heart rate in some participants that lasted for 12 weeks longer.

An advantage of requesting pre-existing data from wearable devices is the ability to create a longitudinal dataset covering a period before enrolment, in some cases for many years. We found that higher historical physical activity was negatively correlated with the development of the passive marker of long COVID—ie, the persistent increased resting heart rate at 12 weeks following COVID-19 diagnosis. To our knowledge, no other study to date has considered the effect of historical activity or fitness level on the risk of developing long COVID, but self-reported historical fitness has been shown to reduce the severity of acute COVID-19.^20^

Sleep duration was not significantly associated with long COVID; however, sleep might be worth further investigation as it has been previously implicated in long COVID,^21^ alongside other markers of historical health in datasets larger than this study. Age was significantly associated with the risk of developing long COVID, in agreement with the symptom-based findings in this study and the findings of multiple other studies.^3^, ^21^, ^22^ Female sex was not a significant risk factor but had a positive coefficient and a relatively low p value, which could indicate an absence of power. However, a similar study exploring resting heart rate also found that female sex was not significantly associated with an increase in risk.^19^ The a-priori decision to use 12 weeks as the long COVID period might have under-represented differences between sexes, given that the following months showed a slower reduction in elevated heart rate among female participants.

The estimated prevalence of long COVID in the literature is diverse. The proportion of people in the group of patients with symptom-based long COVID (12·1%) in this study is within the bounds of similar studies.^9^ Variance could be explained through sociodemographic differences across cohorts, methodological differences in the collection of symptom data, or how long COVID is defined based on collected symptom data.

Our results show that fatigue was the longest lasting symptom, with several participants reporting fatigue for more than 140 days, which is consistent with previously published research.^22^, ^23^ In agreement with the regression on resting heart rate in this study, as well as findings from previous studies,^3^, ^21^, ^22^ age was found to be a significant risk factor for long COVID, with patients older than 40 years at high risk. BMI was not found to be a significant risk factor. Other studies have shown that female sex is positively associated with long COVID,^3^, ^21^, ^22^, ^24^ but we did not find a significant association in this study. This observation might be partly due to the way of identifying long COVID and the period (12 weeks) considered. The change in resting heart rate is similar for men and women at 12 weeks, but appears to remain higher than baseline for female participants while it returns to a baseline level for men between 12 and 24 weeks (figure 1). We found that comorbidities, such as asthma, hypertension, and diabetes, were not significantly associated with risk of developing long COVID in this study; however, this factor had an OR (1·4), which is similar to the results of a 2023 meta-analysis.^25^ An absence of power in the COVID-19 cohort in this study reduces our ability to deduce significance for these sociodemographic and comorbidity factors.

Investigation of metrics from wearable devices and self-reported questionnaires showed that, although not significant (but close to the threshold), PHQ-8 scores at baseline and during the acute COVID-19 phase were positively associated with long COVID. This finding indicates that a period of low mood before and during infection could be a risk factor for long COVID; however, further work is needed to substantiate this. Furthermore, resting heart rate during the acute COVID-19 phase had a non-significant positive associated with long COVID and could be a potential risk factor. We also observed that resting heart rate was persistently increased in the cohort of patients with symptom-based long COVID, which also shows agreement between the two approaches of qualifying long COVID.

There were differences between the analyses of long COVID based on either passive data or active data. Notably, there was a higher number of variables considered in the symptom-based long COVID regression than in the passive-based COVID-19 regression. This observation was largely due to the fewer participants with passive data surrounding and following a diagnosis event, and the further reduction that would have been required to include active data. Therefore, the analysis of passive data on long COVID focused on basic sociodemographic variables and historical data collected passively.

This study brings together self-reported symptoms, data from wearable devices, and frequent mental health surveys in a patient population who are not necessarily hospitalised. Both the symptom-based and passive data approaches showed that age is a risk factor for long COVID. The availability of historical data from wearable devices is unique and has enabled a perspective from participants in the pre-pandemic period. Increased historical activity, which suggests that a participant had engaged in a greater total duration of moderate or greater exercise before COVID-19 diagnosis, was found to be protective against the resting heart rate proxy for long COVID.

The passive data approach uses existing data that are highly available among individuals who own wearable devices, is unbiased by subjective rating and identification of symptoms, and does not burden participants; however, it is limited in symptom scope to what a wearable device can measure. Meanwhile, the approach based on self-reported symptoms allows the reporting of a wider range of symptoms than would be captured through wearable sensors and more concrete labels in the absence of a robust algorithm classifying long COVID by use of passive data.

There are multiple limitations to this study. The two definitions of long COVID used here are weak approximations of a true label. Although we showed group-wide differences in mental health measures and physiological signals during the post-COVID-19 phase, the use of changes in resting heart rate during this period was non-specific and the effect of COVID-19 could have been overwhelmed by natural variability in each individual case. Monitoring of self-reported symptoms requires time and commitment on the part of the person monitoring their COVID-19 recovery, which might be unrealistic to expect, especially given the increased prevalence of depression and fatigue during this period. In both cases, we assumed a consistent deviation from a healthy baseline value. However, symptoms of long COVID can fluctuate or show signs of relapse and remission.^26^

The nature of community-sourced mobile health studies, which rely on self-motivated participation, can lead to sporadic engagement. Completion rates can be influenced by the condition and sociodemographic variables being studied; therefore, self-reported data can rarely be assumed to be missing at random. Furthermore, the open enrolment framework biased the groups participating to those who had the study advertised to them in a way that reached them and who were self-motivated to take part. Additionally, this study only looks at participants with Fitbit devices. Fitbit owners might not represent the general population, or even the larger population of wearable users.

Despite an over-representation of female participants and individuals aged 35–64 years, both variables were controlled for and there was a representation across sexes and age groups. Most participants were from the UK and were White; therefore, findings should not be generalised outside of this population.

Multiple testing was adjusted through correcting the false discovery rate. We believed that the increased power and reduced type 2 error rate were preferable in a largely exploratory study, in which a more stringent correction could lead to an underestimation of harm. The issues of representativeness and significance of results could be addressed through a meta-analysis of similar studies that were conducted across various countries.

The changing nature of the COVID-19 pandemic led to large societal and public health interventions,^27^ many of which would have also influenced mental wellbeing and physical health. For instance, lockdown measures coincided with increased infection rates and influenced physical activity,^28^ sleep duration and quality,^29^, ^30^ resting heart rate,^30^ and mental health.^29^ Therefore, when monitoring recovery from COVID-19 through mobile health technologies, we should also consider the wider context. Time-matching controls help to account for these changes, but the effects of concurrent events should be investigated further.

Looking forward, the pervasive and continuous nature of health data from wearable devices could provide an opportunity for diagnostic and prognostic clinical monitoring of chronic conditions, such as long COVID. Additionally, these data could help us to better reach remote patients and understand disease trajectories and their episodic nature, which might not otherwise be apparent in other clinical data that are collected less frequently and often in-person.

In conclusion, we have shown a measurable difference in measures of mental health and physiological signals from commercial wearable devices between individuals who tested positive for COVID-19 and matched controls during the 16 weeks following the date of COVID-19 diagnosis. We compared two methods of inferring the presence of long COVID, one of which was based on persistent changes in resting heart rate and the other on persistent self-reported symptoms of COVID-19. We explored risk factors of long COVID using demographics, self-reported symptoms, and passive data from wearable devices, and compared them with results from the existing literature base. We found that increased levels of historical activity reduced the risk of developing long COVID. In the future, we plan to assess the feasibility of combining studies to create larger datasets or meta-analyses, to explore interactions between metrics during the acute COVID-19 phase and their effects on recovery, and to investigate the additional effect of public health and safety measures.

### Contributors

### Data sharing

De-identified participant data are available for academic research purposes upon request to the corresponding author and the signing of a data access agreement. A data dictionary, survey responses, data from wearable fitness devices, and sociodemographic data are available.

## Declaration of interests

AAF reports shares in Google, the parent company of Fitbit, which produces the wearable devices used in the study to collect data. No funding or devices were provided by Google or Fitbit, but Fitbit advertised the study in the UK Fitbit app. All other authors declare no competing interests.
