# Supplementary material for: Physiological presentation and risk factors of long COVID in the UK using smartphones and wearable devices: a longitudinal, citizen science, case–control study
Source: Lancet Digit Health. 2024 Aug 12;6(9):e640–50. doi: 10.1016/S2589-7500(24)00140-7 (PMC11832456; doi:10.1016/S2589-7500(24)00140-7)
Supplement: Supplementary appendix [file mmc1.pdf]

THE LANCET
Digital Health

Supplementary appendix

This appendix formed part of the original submission and has been peer reviewed. We post it as supplied by the authors.

Supplement to: Stewart C, Ranjan Y, Conde P, et al. Physiological presentation and risk factors of long COVID in the UK using smartphones and wearable devices: a longitudinal, citizen science, case–control study. *Lancet Digit Health* 2024; published online Aug 12. https://doi.org/10.1016/S2589-7500(24)00140-7.

Table of Contents

Table of collected mobile health metrics 1
Flow chart 2
Group-wide analysis visual summary 3
RHR-defined L-COVID analysis visual summary 3
Groupwide adjusted logistic regression. 4
Control matching algorithm 5
Participant engagement patterns in the first year of enrolment 6
Strobe Checklist 7

Table of collected mobile health metrics

Category	Metric	Frequency	Description
Questionnaires	PHQ-8	14 days	A measure designed to assess the severity of depression[10]
	GAD-7	14 days	A measure designed to assess the severity of generalised anxiety[11]
	Arousal-Valence	Ad-hoc / twice-weekly	A self-rated continuous scale of current happiness and energy, aiming to correspond to emotional valence and arousal [12].
	Symptoms relating to COVID-19	Ad-hoc / twice-weekly	
	Diagnosis	Ad-hoc	Self report diagnosis by Antigen, PCR, Symptom
Fitbit	Heart rate	Daily	Daily resting heart rate provided by Fitbit Web & API.[13]
	Heart rate variability	Daily	Daily root mean square of successive differences (RMSSD) provided by Fitbit Web API[14]
	Sleep duration	Per-sleep	Estimated duration of a sleep[15]
	Sleep efficiency	Per-sleep	Fitbit calculates a score for each recorded sleep based on duration, heart rate, motion, and time spent in detected sleep stages[15].
	Step count	Daily	[15]
	Activity log	Daily	[15]

Table 1 Active and passive mobile health metrics collected in this study.

Flow chart

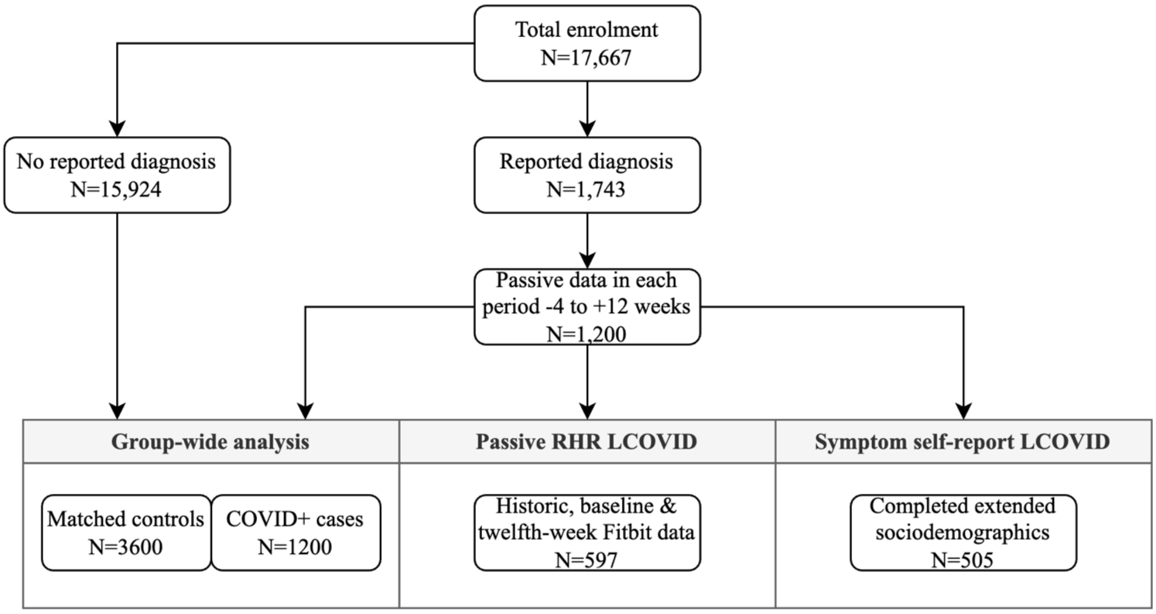

A flow chart showing participants numbers at different stages of the study. The matched controls were included from the no-diagnosis cohort if the matched participant had active self-reported data present at least twelve weeks after the matched date. The rationale was to only include participants who were still actively engaged and therefore likely to have reported a diagnosis if they had been COVID-positive during the study. The selection criteria for the passive RHR LCOVID group is given in the main text.

Group-wide analysis visual summary

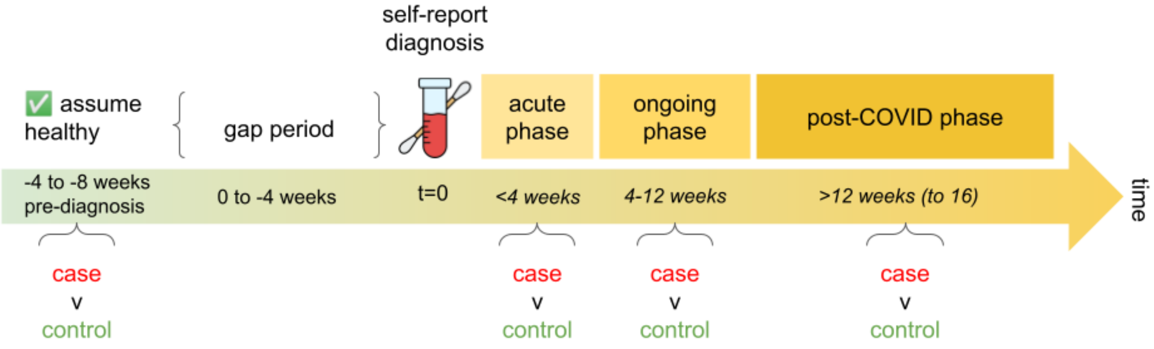

RHR-defined L-COVID analysis visual summary

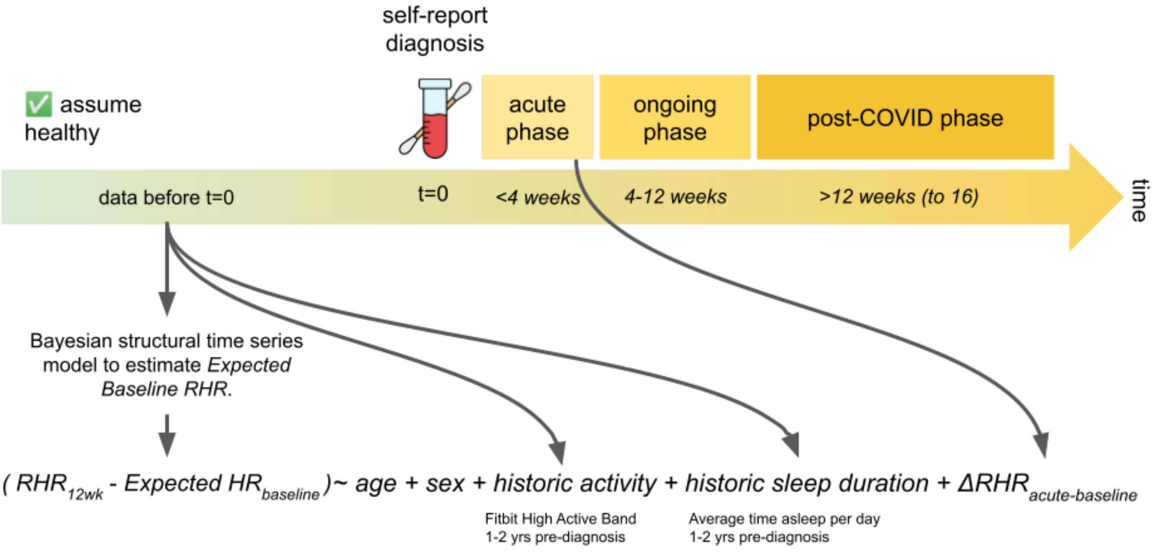

Groupwide adjusted logistic regression.

Metric	Acute			Ongoing			Post		
	**OR**	**95% CI**	**p-value**	**OR**	**95% CI**	**p-value**	**OR**	**95% CI**	**p-value**
RHR (bpm)	1.06016	[1.03432 1.08665]	**<0·0001***	1.10917	[1.08139 1.13766]	**<0·0001***	1.04090	[1.01522 1.06724]	**1.66e-03***
RMSSD	0.99719	[0.736738 1.34972]	0.99	1.09565	[0.81919 1.4654]	0.54	1.00654	[0.674165 1.50277]	0.97
Steps	0.99985	[0.999831 0.999874]	**<0·0001***	0.99996	[0.999944 0.999982]	**1.14e-04***	0.99998	[0.999958 0.999996]	**0.02***
Sleep efficiency	0.99379	[0.982824 1.00487]	0.27	0.99846	[0.987196 1.00984]	0.79	0.99494	[0.984147 1.00585]	0.36
Sleep duration (minutes)	0.99957	[0.998322 1.00082]	0.50	1.00025	[0.999002 1.0015]	0.69	1.00041	[0.999176 1.00164]	0.52
PHQ-8	1.06783	[1.05086 1.08508]	**<0·0001***	1.04553	[1.02557 1.06588]	**<0·0001***	1.03397	[1.01117 1.05728]	**3.32e-03***
GAD-7	1.02452	[1.00641 1.04295]	**7.77e-03***	1.02012	[0.998576 1.04214]	0.07	1.03016	[1.00568 1.05524]	**0.02***
Arousal	0.16237	[0.129536 0.203514]	**<0·0001***	0.51754	[0.404985 0.661368]	**<0·0001***	0.63717	[0.483652 0.839404]	**1.35e-03***
Valence	0.29167	[0.231234 0.367899]	**<0·0001***	0.65344	[0.507303 0.841683]	**9.86e-04***	0.75447	[0.564517 1.00833]	0.06

Control matching algorithm

For each case participant we match 3 control participants.

1. Repeat 3 times:
    a. For each case, match all other participants that fit the following criteria:
        i. Same sex
        ii. Within 2 years of age
        iii. With at least one active task (questionnaire) completed at least 12-weeks after the matched case's date of COVID diagnosis.
    b. Select one random matching participant without replacement.

The requirement for one active task is designed to reduce the likelihood of matching a participant who has dropped out or is inactive and could have contracted an unreported case of COVID-19 while still donating passive data.

Participant engagement patterns in the first year of enrolment

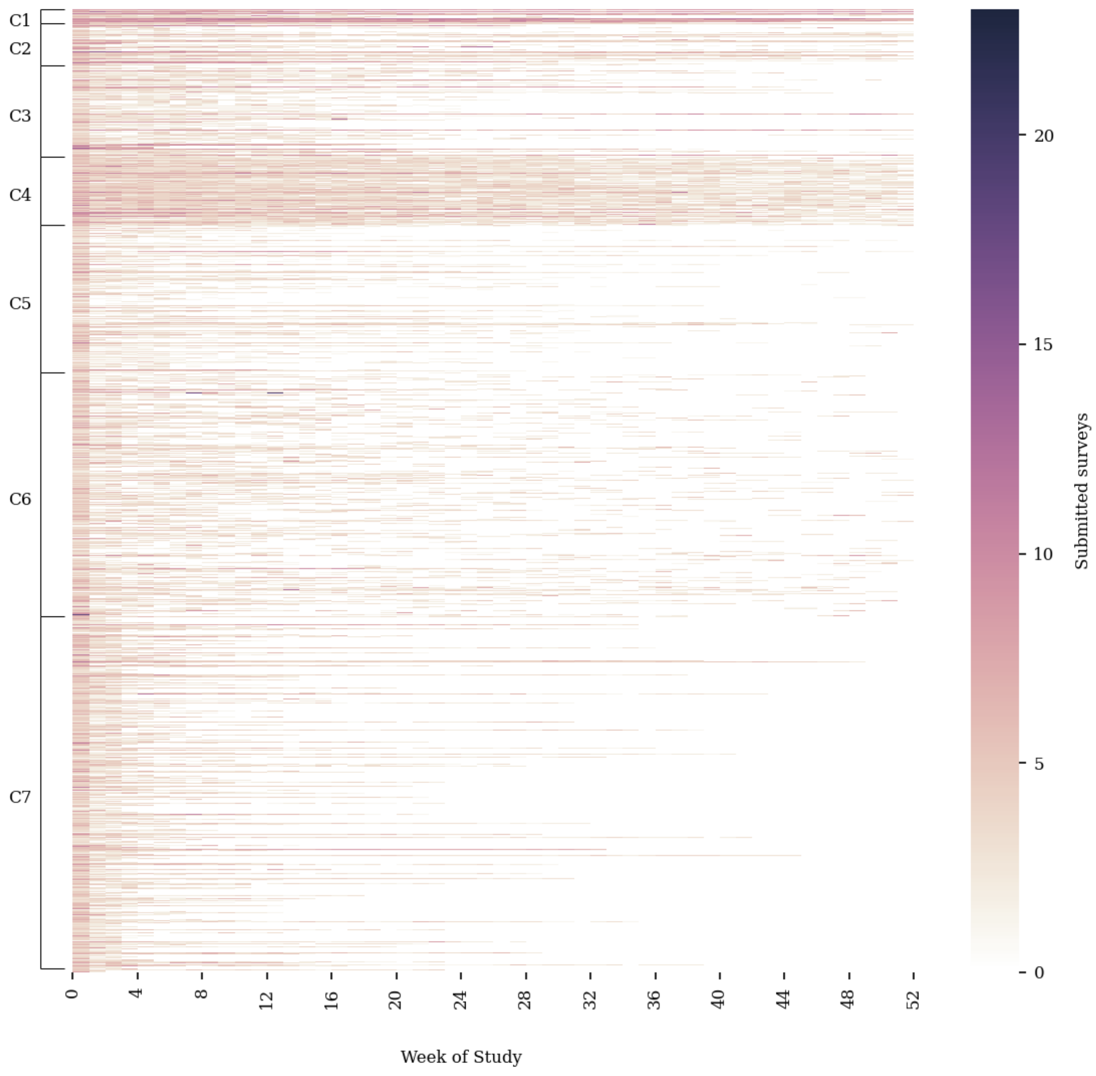

A heatmap showing the number of submitted questionnaires in the first year of enrolment across 11299 participants. Participants were clustered using a hidden Markov model approach to aid visualization of different patterns of engagement.

Strobe Checklist

	Item No	Recommendation	Page number
**Title and abstract**	1	(*a*) Indicate the study's design with a commonly used term in the title or the abstract	1
		(*b*) Provide in the abstract an informative and balanced summary of what was done and what was found	1
**Introduction**			
Background/rationale	2	Explain the scientific background and rationale for the investigation being reported	4
Objectives	3	State specific objectives, including any prespecified hypotheses	4
**Methods**			
Study design	4	Present key elements of study design early in the paper	5
Setting	5	Describe the setting, locations, and relevant dates, including periods of recruitment, exposure, follow-up, and data collection	5
Participants	6	(*a*) Give the eligibility criteria, and the sources and methods of case ascertainment and control selection. Give the rationale for the choice of cases and controls	5
		(*b*) For matched studies, give matching criteria and the number of controls per case	5
Variables	7	Clearly define all outcomes, exposures, predictors, potential confounders, and effect modifiers. Give diagnostic criteria, if applicable	5
Data sources/ measurement	8*	For each variable of interest, give sources of data and details of methods of assessment (measurement). Describe comparability of assessment methods if there is more than one group	5, Appendix p2
Bias	9	Describe any efforts to address potential sources of bias	5, 13
Study size	10	Explain how the study size was arrived at	5
Quantitative variables	11	Explain how quantitative variables were handled in the analyses. If applicable, describe which groupings were chosen and why	6
Statistical methods	12	(*a*) Describe all statistical methods, including those used to control for confounding	6
		(*b*) Describe any methods used to examine subgroups and interactions	-
		(*c*) Explain how missing data were addressed	11
		(*d*) If applicable, explain how matching of cases and controls was addressed	7, Appendix p6
		(*e*) Describe any sensitivity analyses	-
**Results**			
Participants	13*	(a) Report numbers of individuals at each stage of study—eg numbers potentially eligible, examined for eligibility, confirmed eligible, included in the study, completing follow-up, and analysed	5, 9, 11
		(b) Give reasons for non-participation at each stage	
		(c) Consider use of a flow diagram	Appendix p3

Descriptive data	14*	(a) Give characteristics of study participants (eg demographic, clinical, social) and information on exposures and potential confounders	P6
		(b) Indicate number of participants with missing data for each variable of interest	11
Outcome data	15*	Report numbers in each exposure category, or summary measures of exposure	5, 11
Main results	16	(*a*) Give unadjusted estimates and, if applicable, confounder-adjusted estimates and their precision (eg, 95% confidence interval). Make clear which confounders were adjusted for and why they were included	8, 10, 11, Appendix p5
		(*b*) Report category boundaries when continuous variables were categorized	11
		(*c*) If relevant, consider translating estimates of relative risk into absolute risk for a meaningful time period	-
Other analyses	17	Report other analyses done—eg analyses of subgroups and interactions, and sensitivity analyses	10
Discussion			
Key results	18	Summarise key results with reference to study objectives	12, 13
Limitations	19	Discuss limitations of the study, taking into account sources of potential bias or imprecision. Discuss both direction and magnitude of any potential bias	13, 14
Interpretation	20	Give a cautious overall interpretation of results considering objectives, limitations, multiplicity of analyses, results from similar studies, and other relevant evidence	13, 14
Generalisability	21	Discuss the generalisability (external validity) of the study results	14
Other information			
Funding	22	Give the source of funding and the role of the funders for the present study and, if applicable, for the original study on which the present article is based	Abstract, Acknowledgements
